# Ablation of Aquaporin-9 Ameliorates the Systemic Inflammatory Response of LPS-Induced Endotoxic Shock in Mouse

**DOI:** 10.3390/cells10020435

**Published:** 2021-02-18

**Authors:** Angela Tesse, Patrizia Gena, Michael Rützler, Giuseppe Calamita

**Affiliations:** 1 INSERM, CNRS, UNIV Nantes, l’institut du Thorax, 44007 Nantes, France; 2 Department of Biosciences, Biotechnologies and Biopharmaceutics, University of Bari “Aldo Moro”, 70125 Bari, Italy; annapatrizia.gena@uniba.it; 3 Apoglyx AB, c/o Anyo AB, Ideon Science Park, 22370 Lund, Sweden; michael.rutzler@apoglyx.com; 4 Division of Biochemistry and Structural Biology, Department of Chemistry, Lund University, 22100 Lund, Sweden

**Keywords:** membrane transport, hydrogen peroxide, peroxiporins, aquaglyceroporins, LPS, sepsis, inflammation, nitric oxide, superoxide anion, NF-κB pathway, redox signaling, drug targets

## Abstract

Septic shock is the most severe complication of sepsis, being characterized by a systemic inflammatory response following bacterial infection, leading to multiple organ failure and dramatically high mortality. Aquaporin-9 (AQP9), a membrane channel protein mainly expressed in hepatocytes and leukocytes, has been recently associated with inflammatory and infectious responses, thus triggering strong interest as a potential target for reducing septic shock-dependent mortality. Here, we evaluated whether AQP9 contributes to murine systemic inflammation during endotoxic shock. Wild type (*Aqp9^+/+^*; WT) and *Aqp9* gene knockout (*Aqp9^−/−^*; KO) male mice were submitted to endotoxic shock by i.p. injection of lipopolysaccharide (LPS; 40 mg/kg) and the related survival times were followed during 72 h. The electronic paramagnetic resonance and confocal microscopy were employed to analyze the nitric oxide (NO) and superoxide anion (O_2_^−^) production, and the expression of inducible NO-synthase (iNOS) and cyclooxigenase-2 (COX-2), respectively, in the liver, kidney, aorta, heart and lung of the mouse specimens. LPS-treated KO mice survived significantly longer than corresponding WT mice, and 25% of the KO mice fully recovered from the endotoxin treatment. The LPS-injected KO mice showed lower inflammatory NO and O_2_^−^ productions and reduced iNOS and COX-2 levels through impaired NF-κB p65 activation in the liver, kidney, aorta, and heart as compared to the LPS-treated WT mice. Consistent with these results, the treatment of FaO cells, a rodent hepatoma cell line, with the AQP9 blocker HTS13268 prevented the LPS-induced increase of inflammatory NO and O_2_^−^. A role for AQP9 is suggested in the early acute phase of LPS-induced endotoxic shock involving NF-κB signaling. The modulation of AQP9 expression/function may reveal to be useful in developing novel endotoxemia therapeutics.

## 1. Introduction

A major challenge in intensive care medicine is the treatment of sepsis, a critical condition has been defined as a life-threatening organ dysfunction that is caused by a dysregulated host response to infection according to the Sepsis-3 guideline [1]. In modern medicine, sepsis remains associated with high rates of morbidity and mortality and the associated costs in the intensive care units worldwide [2,3].

Gram-negative bacteria are the most common cause of septic shock. The noxious effects of Gram-negative bacteria are triggered by the lipopolysaccharide (LPS), a non-secreted, heat-stable endotoxin [4,5].

Aquaporins (AQPs), a family of membrane channel proteins, are emerging as key players in sepsis exerting roles in inflammation [6], redox-depending signaling [7,8], and in different processes of sepsis [9]. Mammalian AQPs are composed of 13 distinct homologues (AQP0-12) which are subdivided into three main groups: (*i*) *orthodox aquaporins* (AQP0, AQP1, AQP2, AQP4, AQP6, and AQP8), members that were initially believed to transport only water, (*ii*) *aquaglyceroporins* (AQP3, AQP7, AQP9, and AQP10), isoforms allowing for the permeation of small solutes, particularly glycerol, in addition to water, and (*iii*) *superaquaporins* (AQP11 and AQP12), two homologues that are characterized by their distinct evolutionary pathway and intracellular localization, and whose substrate selectivity is still object of debate. However, this classification is not exhaustive since additional solutes can be transported, sometimes even more efficiently than the molecules that were initially considered to be the only substrates. AQP3, AQP6, AQP8, and AQP9 are also permeable to ammonia and they are termed *ammoniaporins* [10]. AQP1, AQP3, AQP5, AQP8, AQP9, and, as recently reported [11], also AQP11 allow the transport of hydrogen peroxide, and, for this biophysical feature, are called *peroxiporins* [12,13,14,15]. Moreover, AQP7 and AQP9 have been reported to carry arsenite, AQP6 to transport nitrate/halide ions and AQP9 to facilitate monocarboxylate transport [16]. Some AQP channels have also been reported to facilitate the transmembrane exchange of physiologically important gases, such as CO_2_, NO, or O_2_ [17,18,19]. Expression, transport properties, and chemical gating [20,21] of AQPs are matter of intense investigation in all areas of the body and a number of important roles, some of them even unexpected [22], have been described, both in health and disease (for review, see [23]).

In the last few years, physiological roles also in the immune system are emerging for AQPs. AQP3 is reported to regulate T cell function and macrophage migration [24], AQP9 is required for CD8^+^ T cell longevity and fast response to rechallenge [25] while AQP5 and AQP9 seem to be involved in neutrophil and macrophage cell migration [26,27,28]. The inflammasome is an important key modulator of the immune response and it affects the immune response by the release of proinflammatory cytokines. AQP expression is altered during inflammation [6,29] and AQP-mediated molecular transport in macrophages seems to be a common element unifying the variety of NLR family pyrin-domain containing protein 3 (NLRP3) inflammasome activators [30,31]. NLRP3 inflammasome is upregulated in sepsis [32]. Therefore, it is not surprising that studies with patients and animal models indicate involvement of AQPs in many dysfunctions in sepsis where their expression appears to be differently regulated, depending on the noxious stimuli, including bacterial infection [for review see ref. 9]. Therefore, the interest about the role of AQPs in sepsis is rapidly growing. Thus, assessing their regulatory mechanisms of differential expression and functional meaning may be of translational value in developing novel sepsis therapeutics.

In rodents, AQP9 is an aquaglyceroporin that is mostly known for its relevance in maintaining the energy balance [33]. In the liver, AQP9 is the primary route through which glycerol is imported from portal blood to hepatocytes during short-term fasting [34,35,36]. As said above, roles for AQP9 have also been reported in the immune system in regulating T cell longevity [25], the establishment of contact hypersensitivity [27], and neutrophil and macrophage cell migration [26]. In a recent in vitro study, AQP9 expression was also found to affect murine bone marrow dendritic cells maturation in response to inflammatory stimulation [37]. Following LPS administration, the absence of AQP9 resulted in a decreased release of pro-inflammatory cytokines and chemokines, including the interleukins (IL) 1α, 1β, 6, and 12, the tumor necrosis factor alpha (TNF-α), the chemokine (C-C motif) ligands 3-5 (CCL3-5), and the keratinocytes-derived chemokine (KC). Furthermore, *Aqp9* knock-down led to a perturbed chemokine receptor switch following LPS treatment.

In this study, we use both a murine model of LPS-induced endotoxemia in the presence or absence of AQP9 (*Aqp9*^−/−^ knockout mice) and a cell model that was treated with LPS, with or without blocking AQP9 to evaluate whether this AQP is involved in the systemic inflammation that is caused by endotoxins and to study the signaling pathways involved.

## 2. Materials and Methods

### 2.1. Animals

*Aqp9*^−/−^ gene knockout (KO) mice that were generated by targeted replacement of part of exon 2 sequence by a 1.7-kb sequence coding for the neomycin phosphotransferase expression cassette were obtained from the Nielsen group [38]. *Aqp9* KO mice were crossed with isogenic C57BL/6J *Aqp9*^+/+^ wild type (WT) mice obtained from Envigo RMS srl (S. Pietro al Natisone, Italy) at regular intervals. The experimental animals were littermates. Mouse genotyping was performed by a standard PCR method, as described in a previous study [38]. Figure S1 reports a representative profile of the PCR bands amplified by genotyping. All of the animals were housed in air-conditioned room with 12/12 h dark–light cycle with free access to a standard laboratory rodent diet (Altromin-Rieper, Vandoies, Italy) and water *ad libitum*. Animal experiments were carried out in accordance with Directive 2010/63/UE and with the approval of the French and Italian animal care and use Committees of the University of Nantes (SBEA of Nantes) and the French Ministry of the Agriculture (authorization n. 2687) and University of Bari (OPBA di Ateneo) and the Italian Ministry of Health (authorization n. 996/2015-PR), respectively.

### 2.2. LPS-Induced Endotoxic Shock

LPS (from *Escherichia coli* 0111:B4; Sigma, St. Louis, MO, USA) was administered intraperitoneally at a dose of 40 mg/kg in AQP9 WT (*Aqp9*^+/+^) and KO (*Aqp9*^−/−^) male mice that were aged 8–12 weeks to induce endotoxic shock. The control mice received an equivalent volume of vehicle represented by physiological saline solution (0.9% NaCl). Survival rates were assessed in the 72 h following the i.p. injection. In another set of experiments mice were sacrificed after 6 h of LPS i.p. administration by cervical dislocation to harvest organs in order to study the systemic inflammatory and oxidative responses to the endotoxic shock. Buprenorphine 0.1 mg/kg was administrated against animal pain.

### 2.3. Cell Culture

Rat hepatoma FaO cells [The European Collection of Authenticated Cell Cultures (ECACC)] were grown in Coon’s modified Ham’s F12 with 10% fetal bovine serum (FBS) until 80% confluence [39]. Cell incubations were made in humidified atmospheric air at 37 °C with CO_2_ added to 5%. Cell monolayers that were composed of about 5 × 10^5^ cells were exposed for 6 h at 37 °C to the culture medium (1% DMSO) containing 25 µM HTS13286, 1 µg/mL LPS or 25 µM HTS13286 plus 1 µg/mL LPS. The basal condition cells were exposed to the culture medium containing 1% DMSO. At the end of the treatment, cell samples of each condition were harvested and handled to prepare the spin traps for the subsequent nitric oxide (NO) and superoxide anion (O_2_^−^) measurements, as reported below.

The cytotoxicity of HTS13286 in FaO cells was evaluated by a MTT (3-(4,5-dimethylthiazol-2-yl)-2,5-diphenyltetrazolium bromide) assay (Abcam, Milan, Italy). FaO cells were seeded in a 96-well plate and then incubated 24 h in humidified atmospheric air at 37 °C with CO_2_ added to 5%. After being treated with the vehicle alone (1% DMSO) or HTS13286 (25 μM in 1% DMSO) for 24 h, the cells were analyzed by adding MTT. The colorimetric absorbance was measured by an iMark^TM^ microplate reader (Biorad, Tokyo, Japan) at 490 nm.

### 2.4. Electronic Paramagnetic Resonance (EPR) Studies for NO and O_2_^−^ Measurements

NO production was evaluated using Fe^2+^ diethyldithiocarbamate (Fe(DETC)_2_) as spin trap. In order to obtain the NO-spin-trap solution, Na-DETC (3.6 mg; Sigma, Saint Quentin Fallavier, France) and FeSO_4_-7H_2_O (2.3 mg; Sigma, France) were separately dissolved under nitrogen gas bubbling in equal 10 mL volumes of ice-cold Krebs–Hepes buffer or distilled water, respectively. The solutions were rapidly mixed to obtain a pale yellow-brown opalescent colloid Fe(DETC)_2_ solution (0.4 mM), which was immediately used to incubate mouse pieces of organs (liver, kidney, aorta, heart, and lung) or FaO cells. After incubation, 45 min. at 37 °C, spin trap was removed, thus the pieces of organs and cells were frozen in liquid nitrogen.

For O_2_^−^ measurements, the pieces of mouse organs (liver, kidney, aorta, heart, and lung) and FaO cells were allowed in a Krebs-Hepes solution containing 500 µM 1-hydroxy-3methoxycarbonyl-2,2,5,5-tetramethylpyrrolidin (CMH; Noxygen, Denzlingen, Germany), 25 µM deferoxamin (Sigma), and 5 µM DETC (Sigma) under constant temperature (37 °C) for 45 min. Subsequently, organs or cells were frozen in liquid nitrogen. All of the samples were analyzed using a table-top x-band spectrometer Miniscope (Magnettech, MS5000, Berlin, Germany). Recordings were made in liquid nitrogen, using a Dewar flask.

For NO determination the instrument settings were 10 mW of microwave power, 1 mT of amplitude modulation, 100 kHz of modulation frequency, 150 s of sweep time, and three scans. Signals were quantified by measuring the total amplitude of the spectra obtained, after correction of baseline. For superoxide anion evaluation, the instrument settings were the same as for the NO measurement, except of the sweep time, which was 60 s. The signals were quantified by calculating the height of the central peak of the spectra, after the correction of baseline, as previously described [40].

### 2.5. Staining and Confocal Microscopy Imaging

Frozen sections of livers, kidneys, aortas, and hearts (7 µm thick) on glass slides were fixed with cold 100% methanol for 5 min. and then incubated (2 h at room temperature) in blocking buffer [(5% non-fat dry milk or 5% bovine serum albumin in phosphate-buffered saline (PBS)]. Tissue sections were then incubated overnight (4 °C) with either a monoclonal murine anti-iNOS or anti-COX-2 (1:50; BD Transduction Laboratories, Null, San Jose, CA, USA), or a polyclonal antibody that was directed against the p65 subunit of the nuclear factor kappa-light-chain-enhancer of activated B cells (NF-κB p65) (1:100; Cell Signaling, Danvers, MA, USA), for iNOS, COX-2 or NF-κB p65 immunostaining, respectively. Three washes were followed by incubation (1 h, at room temperature, in the dark) with the secondary mouse or rabbit fluorescent Alexa fluor-647-conjugated antibody (1:500; Thermo Fisher Scientific, Illkirch, France). Washes and antibody incubation were performed with a 1:10 dilution of BD Perm/Wash^TM^ buffer (BD Biosciences Transduction Laboratories, Null, San Jose, CA, USA). In another set of experiments, frozen sections were used for the in situ detection of O_2_^−^ with the oxidative fluorescent dye dihydroethidine (DHE; Sigma, France) in PBS. DHE oxidizes to EtBr in the presence of O_2_^−^ and it shows a red fluorescence. A Nikon A1-RS inverted laser scanning confocal microscope (Nikon Instruments Europe BV, Amsterdam, Netherlands) was used for the optical sectioning of the tissue. Digital image recording was performed using the NIS element software (Nikon Instruments Europe BV, Amsterdam, Netherlands). The images were analyzed and processed by ImageJ-win32 software (https://imagej.net/ (accessed on 17 February 2021)) by merging the red fluorescence of iNOS, COX-2, NF-κB p65/Rel A or DHE and the blue fluorescence of nuclei stained with DAPI. For aortas, the green autofluorescence of elastin was also merged to better localize the inflammatory protein immunostaining or oxidative stress staining in the vascular wall. The presence of the NF-κB subunit p65/Rel A within cell nuclei was evidenced by the white spots that the software that were interpreted as co-localization of red and blue staining.

Semi-quantitative analysis of liver, kidney, aorta, and heart iNOS and COX-2 immunostainings was performed by ImageJ-win32 software evaluating the red fluorescence of the confocal microscopy images that were acquired from the sections of the organs harvested from the WT or KO mice sacrificed 6 h after i.p. injection of saline solution or LPS (40 mg/kg). Red fluorescence was expressed in arbitrary units.

### 2.6. Data Analysis

The results of the experiments with mice were expressed as a mean ± SD of n, where n represents the number of animals or plate wells with FaO cells used for each experiment. The survival curves were compared with a log-rank test. The levels of NO and O_2_^−^ were compared using a one-way analysis of variance, followed by a Bonferroni multiple comparison *post-hoc* test. In all cases, a *p* value of less than 0.05 was considered to be significant.

## 3. Results

### 3.1. Aqp9 Deletion Improves Survival of Mice after LPS Exposure

**Figure 1:**
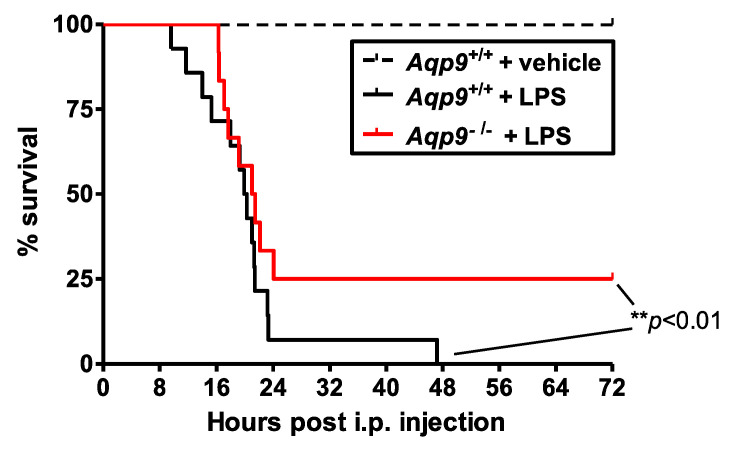
Effect of AQP9 ablation on survival rate in lipopolysaccharide (LPS)-induced sepsis in mice. Survival curves of *Aqp9*^+/+^ (wild type (WT)) mice after i.p. injection of saline solution (dotted black line, n = 6) or LPS (40 mg/kg) (solid black line, n = 14) and *Aqp9*^−/−^ (knockout (KO)) mice after i.p. injection of LPS (solid red line) (n = 12; ** *p* < 0.01).

The relevance of AQP9 in septic shock was evaluated by assessing the effect of the injection of a lethal dosage of LPS, a bacterial endotoxin inducing severe systemic inflammation, on mouse survival during the following 72 h. Only 7% of *Aqp9*^+/+^ mice survived after 24 h and all of the *Aqp9*^+/+^ mice were dead before 48 h, while about 25% of *Aqp9*^−/−^ mice survived after 72 h of LPS challenge (Figure 1). All of the control *Aqp9*^+/+^ animals receiving only physiological saline solution survived to the survival test.

### 3.2. Deletion of AQP9 Protein Protects Against LPS Induced Inflammation

In order to study the involvement of AQP9 in the early phase of systemic inflammation during endotoxic shock, in another set of experiments, the mice were sacrificed at 6 h after the injection of LPS (or saline solution) and lungs, liver, kidneys, aorta, and heart were collected to measure the NO production, iNOS, and COX-2 expression. The 6-h time point after the injection of LPS (or saline solution) was chosen close to, but safely before, some of the animals died, so that the events leading to death (as shown in Figure 1, most LPS-treated WT mice die after 12–20 h of LPS) could be studied without losing some of the experimental mice that would be expected to be the mice with the clearest phenotype.

**Figure 2:**
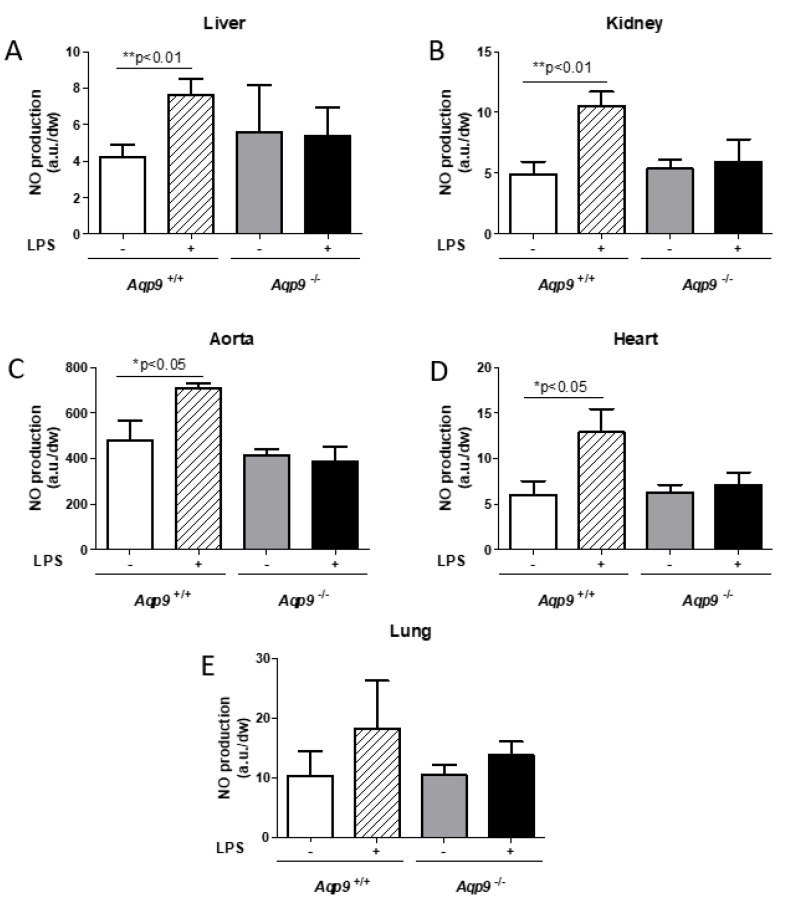
Analysis of inflammatory nitric oxide (NO) production. Electronic paramagnetic resonance measurement of the NO levels of liver (**A**), kidney (**B**), aorta (**C**), heart (**D**), and lung (**E**) of *Aqp9*^+/+^ or *Aqp9*^−/−^ mice 6 h after i.p. injection of physiological saline solution or LPS (40 mg/kg) (see *Materials and Methods* for details) (3–4 animals/group). * *p* < 0.05, ** *p* < 0.01 *Aqp9*^+/+^ vs. *Aqp9*^+/+^ +LPS. *a.u.*, arbitrary units; *dw*, dry weight.

As expected, a significant increase of NO was observed in the liver, kidney, aorta, and heart harvested from WT mice treated with LPS as compared to the control WT animals injected with the vehicle alone due to the noxious insult induced by LPS (Figure 2A–D). Interestingly, when compared to vehicle controls, LPS treatment did not increase NO levels in any of the tested *Aqp9*^−/−^ mouse tissues, i.e., liver, kidney, aorta, heart, and lung (Figure 2A–E). These data were fully consistent with the immunocytochemical analysis of iNOS expression and the localization in the liver, kidney, aorta, and heart of the analyzed mouse specimens. *Aqp9*^+/+^ mice that were challenged with LPS showed much higher levels of iNOS staining than the control WT animals only receiving the vehicle. No significant increase in NO production was seen in the lungs of both LPS-injected WT and KO mice when compared to the control animals that only received the vehicle (Figure 2E).

**Figure 3:**
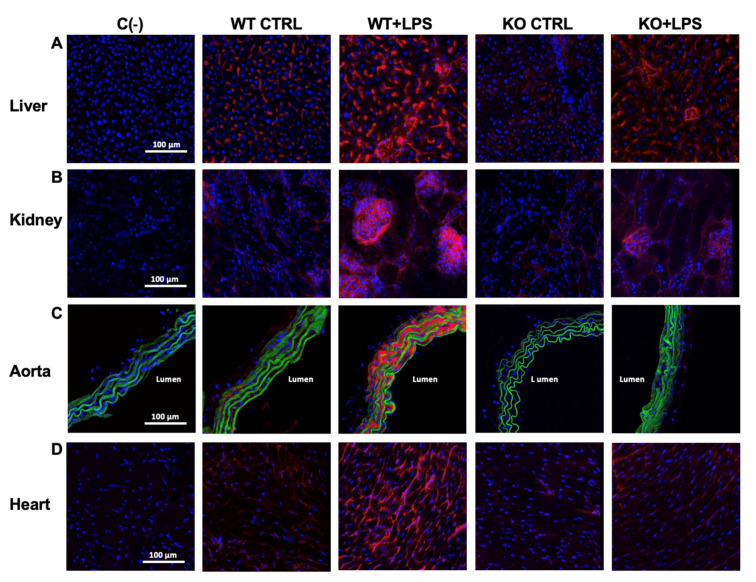
Immunocytochemical analysis of inducible NOS (iNOS) expression. iNOS immunoreactivity (red staining) of liver (**A**), kidney (**B**), aorta (**C**), and heart (**D**) harvested from *Aqp9*^+/+^ (WT) or *Aqp9*^−/−^ (KO) mice sacrificed 6 h after i.p. injection of saline solution (CTRL) or LPS (40 mg/kg) (4 animals/group). The green fluorescence seen in aortas corresponds to elastin autofluorescence. Nuclei are stained by DAPI (blue fluorescence). C(−), negative (no primary antibody).

**Figure 4:**
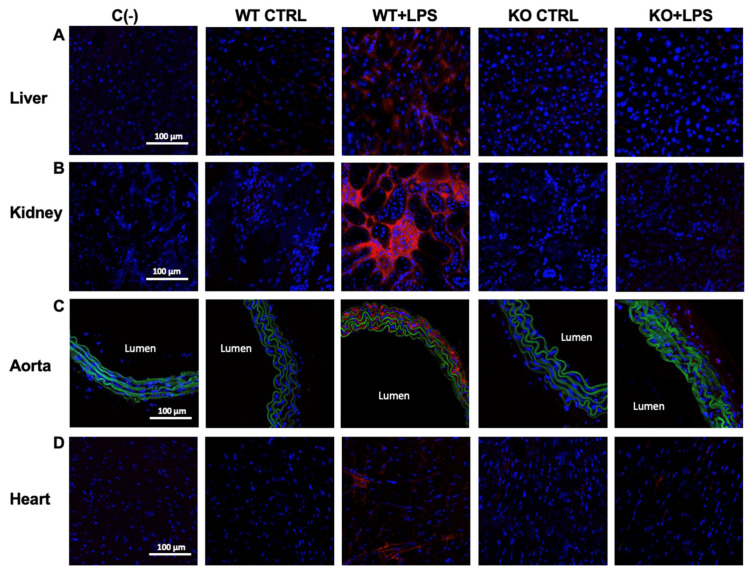
Immunohistochemical analysis of inducible COX (COX-2; red fluorescence) in liver (**A**), kidney (**B**), aorta (**C**), and heart (**D**) from untreated (saline solution) *Aqp9*^+/+^ or *Aqp9*^−/−^ mice (WT CTRL and KO CTRL, respectively) or LPS-treated (40 mg/kg) WT or KO mice (WT+LPS and KO+LPS, respectively) (four animals/group), 6 h after the i.p. injection. The green fluorescence seen in aortas corresponds to elastin autofluorescence. Cell nuclei were stained by DAPI (blue fluorescence). C(−), negative controls (no primary antibody).

iNOS immunoreactivity in the organs of the *Aqp9*^−/−^ mice that were treated with LPS was slightly higher when compared to the control KO animals but significantly lower than that of the LPS-injected WT mice (Figure 3A–D; Table 1). Accordingly, the levels of COX-2, another inflammatory inducible protein, were increased in the liver, kidney, aorta, and heart of the *Aqp9^+/+^* LPS mice, whereas weak or no detectable immunolabeling was found in the corresponding organs harvested from the *Aqp9*^−/−^ LPS-treated animals (Figure 4A–D; Table 1).

### 3.3. Aqp9 Deletion Reduces NF-κB p65 Elevation by LPS

**Figure 5:**
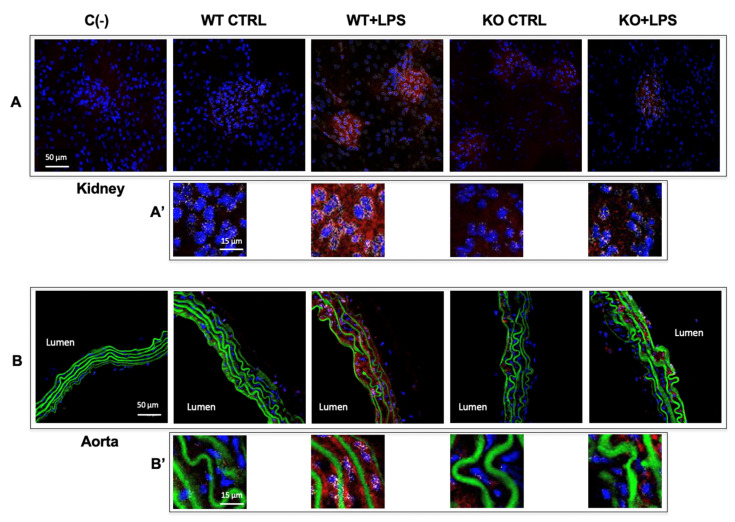
Immunohistochemical analysis of the p65/Rel A subunit of NF-κB in kidney (**A**,**A’**) and aorta (**B**,**B’**) from untreated (saline solution) *Aqp9*^+/+^ or *Aqp9*^−/−^ mice (WT CTRL and KO CTRL, respectively) or LPS-treated (40 mg/kg) WT or KO mice (WT+LPS and KO+LPS, respectively) (four animals/group), 6 h after the i.p. injection. The red fluorescence seen in the cells is related to the cytoplasmic localization of p65/Rel A. Cell nuclei are visualized by DAPI (blue fluorescence). The white spots seen in cell nuclei correspond to the overlapping of DAPI fluoresce and p65/Rel A immunostaining. The green fluorescence in aortas corresponds to elastin autofluorescence. C(−), negative (no primary antibody). The number of white spots seen in the cell nuclei of kidney and aorta of KO+LPS animals appears to be considerably lower than that of WT+LPS animals (**A’**,**B’**).

**Figure 6:**
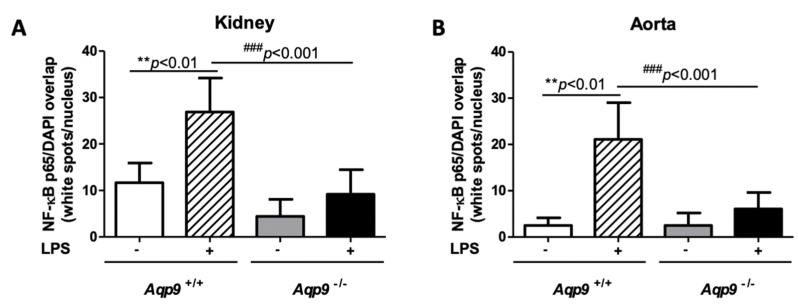
Immunofluorescence evaluation of NF-κB p65 in cell nuclei. The number and intensity of *white spots* corresponding to the DAPI/p65 overlap in cell nuclei was evaluated by the ImageJ-win32 software using confocal microscopy images of kidneys (**A**) and aortas (**B**) harvested from *Aqp9*^+/+^ (WT) or *Aqp9*^−/−^ (KO) mice sacrificed 6 h after the injection of saline solution (control) or LPS (40 mg/kg) (4 animals/group) (see *Materials and Methods* for details). In WT mice, LPS treatment induced a significant increase of the NF-κB p65 extent compared to WT control mice (** *p* < 0.01 *Aqp9*^+/+^ vs. *Aqp9*^+/+^+LPS). No significant increase of the intranuclear level of NF-κB p65 was seen in the kidney and aorta of KO mice that were treated with LPS as compared to the KO control animals (^###^ *p* < 0.001).

Inflammation that is associated with iNOS and COX-2 expressions is regulated by the transcription factor NF-κB p65 [41]. Thus, we decided to investigate the expression of the p65 subunit of this nuclear factor by immunofluorescence labeling. Under inflammatory stimuli triggered by extracellular signals, such as LPS, p65 translocates into the nucleus, where it binds to target genes inducing the transcription of genes coding for several pro-inflammatory proteins [42]. The depletion of AQP9 was associated to a reduced expression of the inflammatory transcription factor NF-κB p65, since weak immunostaining and reduced translocation were seen in the nuclei in the kidney and aorta tissues harvested from the LPS-treated *Aqp9*^−/−^ mice as compared to the respective organs from the endotoxic *Aqp9^+/+^* mice (Figure 5A,A’,B,B’). In both kidneys and aorta, the extent of p65/Rel A immunoreactivity measured into the nuclei of LPS-treated KO specimens was significantly lower than that of the WT counterpart (Figure 6A,B). This profile suggests that the lower mortality in LPS treated *Aqp9*^−/−^ mice involve reduced NF-κB p65 expression and activity.

### 3.4. Lack of AQP9 Protects Against LPS Induced Oxidative Stress

Because LPS is also reported to induce systemic oxidative stress we decided to assess the superoxide anion production during the endotoxic shock in WT and *Aqp9* KO mice.

**Figure 7:**
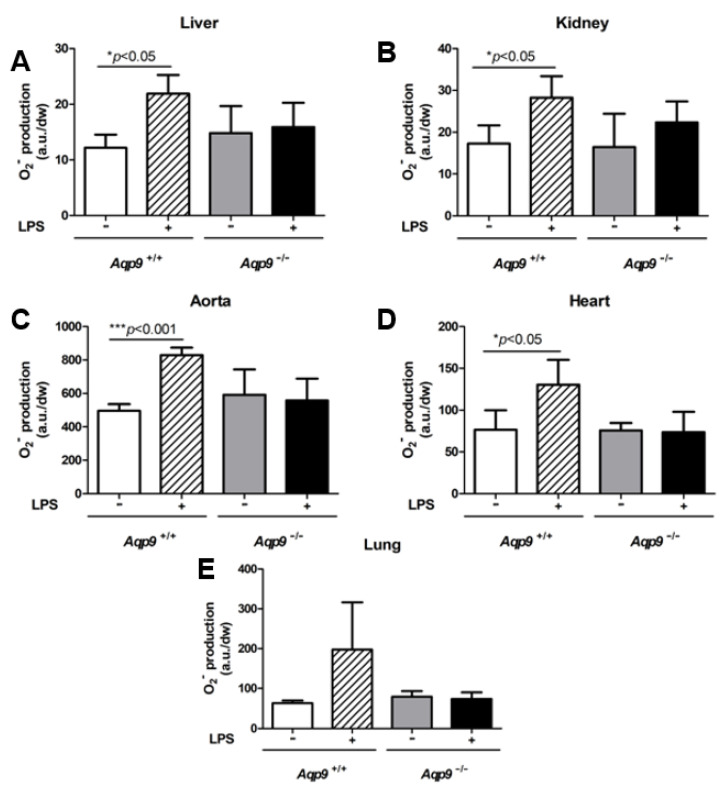
Analysis of superoxide anion (O_2_^−^) production. Electronic paramagnetic resonance measurements of O_2_^−^ in liver (**A**), kidney (**B**), aorta (**C**), heart (**D**), and lung (**E**) of *Aqp9*^+/+^ (WT) or *Aqp9*^−/−^ (KO) mice receiving i.p. injection of saline solution alone or LPS (40 mg/kg *i.p*) for 6 h (see *Materials and Methods* for details) (n = 3–4 animals/group). * *p* <0.05; *** *p* < 0.001, *Aqp9*^+/+^ vs. *Aqp9*^+/+^ +LPS. a.u., arbitrary units; dw, dry weight.

**Figure 8:**
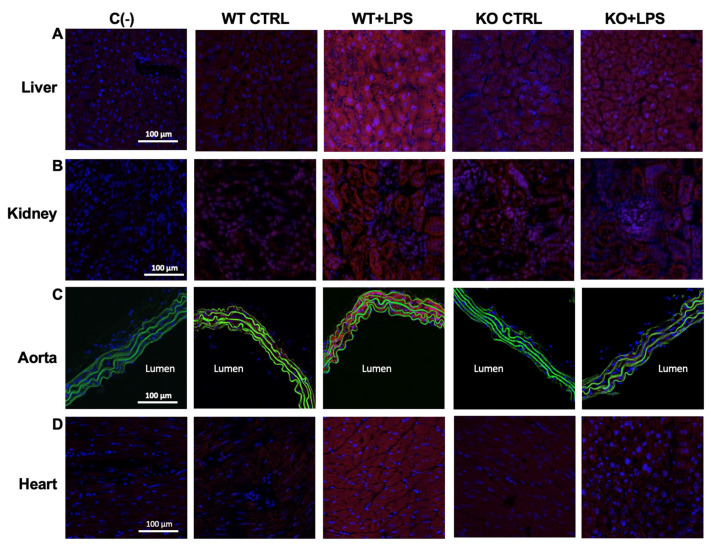
Detection of superoxide anion by dihydroethidine (DHE) (red fluorescence) in liver (**A**), kidney (**B**), aorta (**C**), and heart (**D**) from *Aqp9*^+/+^ or *Aqp9*^−/−^ mice after 6 h of i.p. injection of saline solution (WT CTRL and KO CTRL, respectively) or LPS (40 mg/kg) WT or KO mice (WT+LPS and KO+LPS, respectively) (four animals/group). The green fluorescence seen in aortas corresponds to elastin autofluorescence. Cell nuclei are stained by DAPI (blue fluorescence). C(−), negative controls (no DHE).

As expected, the O_2_^−^ measurements that were obtained using CMH as spin-probe (see *Materials and Methods*) showed a significant increase of superoxide anion in the liver, kidney, aorta, and heart of *Aqp9^+/+^* WT mice, following LPS treatment (Figure 7A–D). This increase was not observed in *Aqp9*^−/−^ mice (Figure 7A–D). This result was confirmed by considerably higher levels of oxidized DHE (Figure 8; red staining), a fluorescent dye detecting O_2_^−^ in LPS-treated *Aqp9^+/+^* WT mice when compared to the respective *Aqp9*^−/−^ mice. No significant changes in O_2_^−^ production were seen between the lungs of the KO and WT animals receiving LPS (Figure 7E).

### 3.5. Inhibition of AQP9 in Rat Hepatoma Cells Reduces the LPS-Induced NO and Superoxide anion Production

FaO cells, a rodent hepatoma cell line expressing AQP9 [43], were utilized to further investigate these observations. FaO cells were challenged with LPS and HTS13286, a selective and potent inhibitor of AQP9 [34] that was utilized to study the role of AQP9 in NO and O_2_^−^ production after LPS exposure.

**Figure 9:**
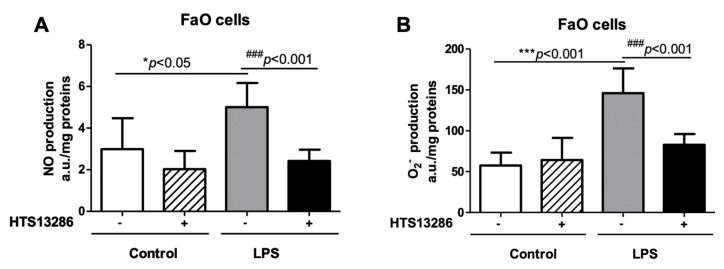
Nitric oxide (NO) (**A**) or superoxide anion (O_2_^−^) measurements (**B**) by electronic paramagnetic resonance in FaO cells incubated or not for 6 h with LPS (1 µg/mL) in absence or in presence of the AQP9 selective inhibitor HTS13286 (25 µM) (see *Materials and Methods* for details). * *p* < 0.05, *** *p* < 0.001 control cells vs. LPS-treated cells in absence of HTS13286; ^###^ *p* < 0.001, LPS-treated cells in absence of HTS13286 vs. LPS-treated cells that were exposed to HTS13286; n = 5–6 experiments for each condition.

As expected, LPS increased the levels of both NO and O_2_^−^ in the FaO cell specimens that were treated with LPS when compared to the vehicle treated control cells (Figure 9A,B). Interestingly, the inhibition of AQP9 by HTS13286 prevented the increase of both free radicals in the cells that were treated with LPS (Figure 9A,B), suggesting the involvement for AQP9 in the signaling pathways stimulated by the endotoxin. HTS13286 did not show any toxic effect on FaO cells (Figure S2).

## 4. Discussion

Here, we provide evidence for an important role of AQP9 in LPS-induced endotoxic shock. *Aqp9^−/−^* mice showed significantly improved survival after LPS administration. The ablation of AQP9 exerted an anti-inflammatory effect with significant attenuation of the inflammatory NO and O_2_^−^ production, following LPS challenge. Furthermore, we observed at the protein level reduced expression of NF-κB p65, and its transcriptionally dependent inflammatory markers, iNOS and COX-2 [44]. In general, many additional pro-inflammatory mediators controlled by NF-κB p65 are known (e.g., TNF-α, IL-1β, IL-6 and MCP-1 [45]) and their contribution to the observed effects of *Aqp9* deletion on the survival to endotoxemia can be anticipated. However, it seems likely that the reduced production of the vasodilator NO may, in part, explain the increased survival of *Aqp9^−/−^* mice in endotoxemia.

Importantly, the effects of *Aqp9* deletion on NO and O_2_^−^ production at the whole organism level could be confirmed in FaO rodent hepatoma cells. There, the LPS-induced elevation of NO and O_2_^−^ was prevented by blocking AQP9 with the selective inhibitor HTS13286 [34].

While important functions of AQP9 in leukocytes during endotoxemia cannot be ruled out, these results suggest that at least part of the altered LPS response in *Aqp9^−/−^* mice is due to AQP9 functions in hepatocytes, a major expression site of AQP9 in rodents and humans e.g., [38,46].

All of the functional components of the canonical LPS receptor, the toll-like receptor 4 (TLR-4), including the CD14 and MD2 co-receptors, have been detected in hepatocytes [47,48,49], and LPS activation of MAPK signaling, NF-κB nuclear translocation, and activation of acute phase protein production have been described [47,48]. Previous work showed that LPS exerts a strong synergistic effect with cytokines, but it was not alone sufficient to induce iNOS expression in primary rat hepatocytes [50]. This suggests that FaO cells may already be in a primed state, which allows enhanced NO production in response to LPS alone.

A working model is proposed to explain how AQP9 intervenes in the early acute phase of the inflammatory reactions that are triggered by LPS in endotoxemia.

In analogy to AQP involvement in several recently discovered cell signaling pathways, LPS binding to a combination of its cognate co-receptors CD14, MD2, and TLR-4 [51,52,53,54], may lead to the activation of a plasma membrane localized member of the NADPH oxidase (NOX) superfamily (e.g., NOX2) [55]. In this model, H_2_O_2_, which results from dismutation (e.g., through extracellular superoxide dismutase-3, SOD3) of NOX generated O_2_^−^, may enter cells through AQP9 acting as second messenger to inactivate a protein-tyrosine phosphatase (PTP) at an active site cysteine [56]. This PTP typically functions as a negative regulator of a tyrosine kinase (TK) signal. Thus, the inactivation of PTP by AQP9-facilitated H_2_O_2_ uptake may result in the amplification of the TK signal and consequent enhancement of NF-κB pathway stimulation and related functional output.

It is known that AQP9 functions as a peroxiporin [57]. Examples for AQP modulation of the NF-κB pathway include AQP3 dependent NF-κB activation in keratinocytes during psoriasis [58], in LPS-induced inflammation in macrophages during liver injury [59], and in B-cell activation [60]. Interestingly, in the latter example, a role for AQP8 in LPS stimulated tyrosine phosphorylation downstream of TLR-4 has been demonstrated, while canonical TLR signaling relies on serine/threonine kinases [61]. AQP-facilitated uptake of extracellular H_2_O_2_ has, at this point, been invoked in several additional downstream signaling cascades [7,12,24,62,63,64,65,66].

The mechanism of AQP9 involvement in inflammatory responses may be different, depending on the type of the noxious stimulus. A role for AQP9 in the extracellular signal-regulated kinase1/2 pathway was suggested in the inflammatory response that is associated with myocardial infarction in rats [67]. Patients with systemic inflammatory response syndrome (SIRS) have enhanced AQP9 expression in activated polymorphonuclear leukocytes as compared to healthy control subjects [68]. A function for AQP9 in human macrophage motility, migration, and phagocytosis was suggested after observing that Gram-negative bacteria, such as *P. aeruginosa*, induce enhanced expression of AQP9 and re-organization in primary monocyte-derived macrophages accompanied by changes in cell size and morphology [26]. Because AQP9 features broad selectivity, its physiological involvement in inflammatory responses may be as pleiotropic as the channel’s function, carrying, among others, water, H_2_O_2_, and glycerol.

Immune cell migration is an important mechanism that occurs in sepsis [69,70] and another potential mechanism linking AQP9 to survival in sepsis may relate to the suggested implication of this AQP in neutrophil cell migration [71]. Neutrophils always appear at an early stage of infection and decreased neutrophil cell migration to the infected organs has been associated with a bad outcome of sepsis, due to incomplete bacterial eradication [72]. However, neutrophils secrete large amounts of proteases and reactive oxygen species, which not only kill bacteria, but also damage host tissues [73]. Hence, an exceedingly neutrophil cell migration to the tissues can cause multiple organ failure, leading to death [73]. AQP5 expression in mouse and human neutrophils has been suggested to influence survival following LPS by altering cell migration [28]. The potential role of AQP9 in neutrophil cell migration in tissues after septic insults is a matter for future studies.

## 5. Conclusions

Altogether, our results suggest a role of AQP9 in the early stage of LPS-induced endotoxic shock, involving the NF-κB pathway, potentially through facilitating uptake of extracellular H_2_O_2_, and PTP dependent signaling. However, further work is needed to fully understand the AQP9 dependent signal transduction in hepatocytes, as well as the potential involvement of other cell types, such as neutrophils, in the LPS-induced systemic inflammatory reaction in mice.

## Figures and Tables

**Table 1:** Red fluorescence semi-quantitative evaluation of iNOS and COX-2 immunostaining.

		WT CTRL	WT + LPS	KO CTRL	KO + LPS
iNOS (a.u.)	liver	4.48 ± 1.23	26.97 ± 5.23 ***	3.32 ± 0.32	7.69 ± 0.96 ^###, ++^
	kidney	3.07 ± 1.03	50.56 ± 16.39 ***	1.79 ± 0.33	9.63 ± 1.49 ^##^
	aorta	1.16 ± 0.50	69.35 ± 14.02 ***	1.43 ± 0.27	1.26 ± 0.35 ^###^
	heart	2.42 ± 0.40	20.37 ± 2.80 ***	1.98 ± 0.42	4.53 ± 1.17 ^###, +^
COX-2 staining (a.u.)	liver	0.17 ± 0.07	8.37 ± 1.88 ***	0.82 ± 0.80	0.44 ± 0.18 ^###^
	kidney	0.014 ± 0.01	27.31 ± 6.00 ***	0.02 ± 0.01	0.02 ± 0.004 ^###^
	aorta	0.29 ± 0.19	15.80 ± 2.84 ***	0.50 ± 0.17	3.37 ± 0.44 ^###^
	heart	0.10 ± 0.11	18.49 ± 6.62 ***	0.95 ± 0.27	1.68 ± 1.34 ^###^

*** *p* <0.001, WT + LPS vs. WT CTRL; ^##^ *p* < 0.01, ^###^ *p* < 0.001, KO + LPS vs. WT + LPS; ^+^ *p* < 0.05, ^++^ *p* < 0.01, KO CTRL vs. KO + LPS; a.u., arbitrary units.

## Data Availability

No new data were created or analyzed in this study. Data sharing is not applicable to this article.

## Supplementary Materials

The following are available online at https://www.mdpi.com/2073-4409/10/2/435/s1, Figure S1: Representative agarose gel showing the cDNA fragments amplified by PCR in the genotyping of wild type (*Aqp9*^+^/^+^) and Aqp9 gene knockout (*Aqp9*^−^/^−^; KO) mice; Figure S2: Effect of HTS13286 on the viability in FaO cells.
