# Supplementary material for: Ablation of Aquaporin-9 Ameliorates the Systemic Inflammatory Response of LPS-Induced Endotoxic Shock in Mouse
Source: Cells. 2021 Feb 18;10(2):435. doi: 10.3390/cells10020435 (PMC7922179; doi:10.3390/cells10020435)
Supplement: Supplementary file 1 [file cells-10-00435-s001.pdf]

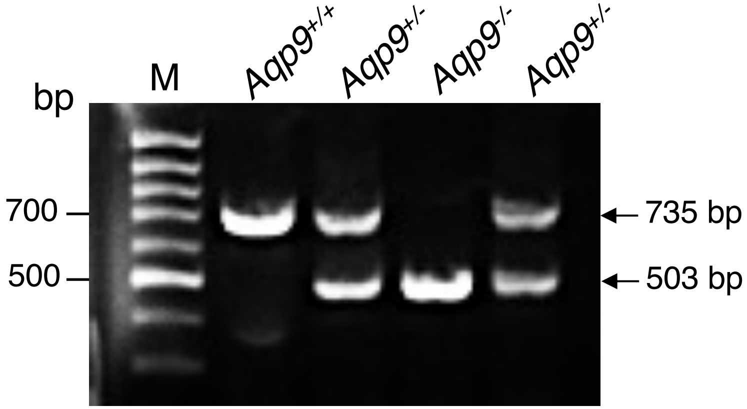

Figure S1. Representative agarose gel showing the cDNA fragments amplified by PCR in the genotyping of wild type (*Aqp9*$^{+/+;}$ WT) and *Aqp9* gene knockout (*Aqp9*$^{-/-}$; KO) mice according to the procedure followed in a previous work by Rojek and coworkers [38]. The wild type genotype is indicated by the amplification of a 735 bp cDNA fragment while the KO genotype leads to a 503 bp DNA fragment. Heterozygous *Aqp9*$^{+/-}$ mice are indicated by the presence of both DNA bands.

Supplementary Figure S1

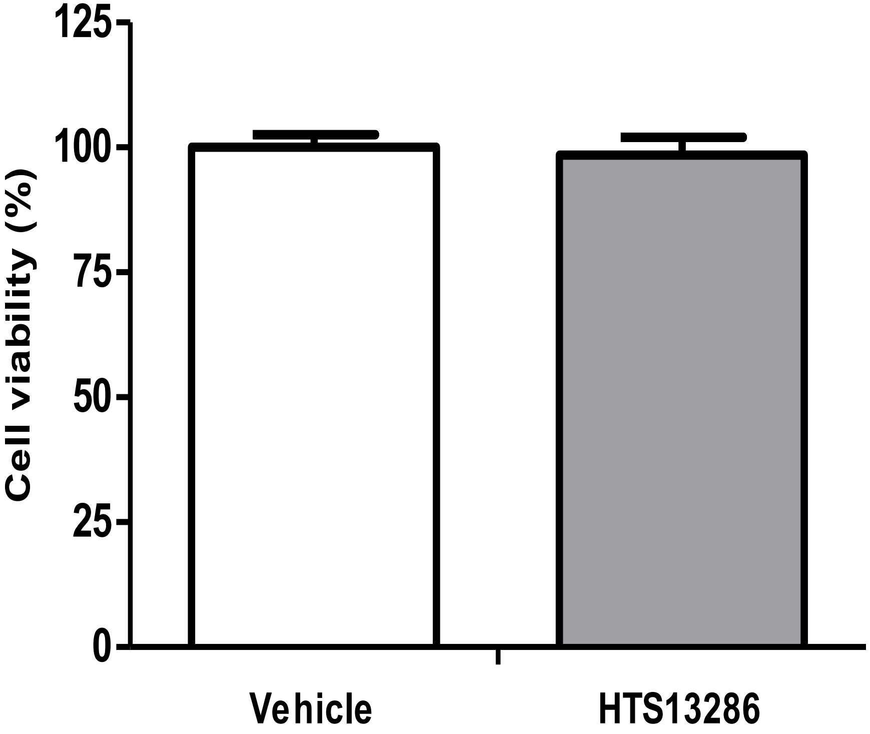

Figure S2. Effect of HTS13286 on the viability in FaO cells. Cultured FaO cells were treated with 1% DMSO (vehicle) or 25 μM HTS13286 (HTS13286) for 24 h. Cell viability was measured by MTT assay. Values are presented as mean ± SD; n = 3.

Supplementary Figure S2
